# Integrated multi-omics reveals novel microbe-host lipid metabolism and immune interactions in the donkey hindgut

**DOI:** 10.3389/fimmu.2022.1003247

**Published:** 2022-11-18

**Authors:** Yan Li, Qingshan Ma, Xiaoyuan Shi, Guiqin Liu, Changfa Wang

**Affiliations:** Shandong Engineering Technology Research Center for Efficient Breeding and Ecological Feeding of Black Donkey, College of Agronomy, Liaocheng University, Liaocheng, China

**Keywords:** multi-omics, donkeys, hindgut, microbe, lipid metabolism, immune

## Abstract

Evidence has shown that gut microbiota play a key role in host metabolism and health; however, little is known about the microbial community in the donkey hindgut as well as the interactions that occur between gut microbes and the host. This study aimed to explore the gut microbiome differences by analyzing the microbial community and differentially expressed genes (DEGs) related to lipid metabolism and the immune system along the donkey hindgut. The hindgut tissues (cecum, ventral colon, and dorsal colon) were separated, and the contents of each section were collected from six male donkeys for multi-omics analysis. There were significant differences in terms of dominant bacteria among the three sections, especially between the cecum and dorsal colon sites. For instance, species belonging to *Prevotella* and *Treponema* were most abundant in the cecum, while the *Clostridiales_bacterium*, *Streptococcus_equinus*, *Ruminococcaceae_bacterium*, etc., were more abundant in the dorsal colon. Apart from propionate, the concentrations of acetate, isobutyrate, valerate and isovalerate were all lower in the cecum than in the dorsal colon (p < 0.05). Furthermore, we identified some interesting DEGs related to lipid metabolism (e.g., *ME1*, *MBOAT1*, *ACOX1*, *ACOX2* and *LIPH*) and the immune system (e.g., *MUC3B*, mucin-2-like, *IL17RC*, *IL1R2*, *IL33*, *C1QA*, and *MMP9*) between the cecum and dorsal colon and found that the PPAR pathway was mainly enriched in the cecum. Finally, we found a complex relationship between the gut microbiome and gene expression, especially with respect to the immune system, and combined with protein-protein interaction (PPI) data, suggesting that the PPAR pathway might be responsible, at least in part, for the role of the hindgut microbiota in the donkeys’ gut homeostasis. Our data provide an in-depth understanding of the interaction between the microbiota and function in the healthy equine hindgut and may also provide guidance for improving animal performance metrics (such as product quality) and equine welfare.

## Introduction

The equine gastrointestinal (GI) tract can be divided into 2 large parts, namely, the foregut and the hindgut (1). The equine hindgut (including the cecum and colon) constitutes approximately 60% of the GI tract (2) and is where the majority of the microbial population (3), which plays an important role in nutrition metabolism and the immune system, is found. Thus, by better understanding the donkey hindgut microbial ecology, we can inform interventions that can improve animal production performance and host health (4). Previous studies of the equine hindgut microbiome have revealed that the taxonomic composition changes of this bacterial community vary with many factors, including age, breed, diet and disease status (5–10). However, knowledge about the hindgut microbial ecosystem and metabolism is still lacking despite being essential to host metabolism and health. A previous study of the GI tract microbiota of Dezhou donkeys conducted in our lab found significant differences in microbial diversity and predicted function between the small and large intestines (1), and did not specifically detect the variation in microbial profiles and function or their interaction with the host in separate segments of the donkey hindgut. In recent years, metagenomics has become a popular method to study microbial communities when we cannot separate one microbe from another (11); this approach is used extensively in animal husbandry and to study its relationship to host health and disease. To better understand the contribution of the equine hindgut microbiome to host metabolism and intestinal homeostasis, it is necessary to provide comprehensive knowledge of its bacterial composition and their functions using the metagenomics method.

The gut microbiota is well known to influence and communicate with the host *via* some signaling metabolites, including bacteria-derived metabolites (such as organic acids), bacterial components and microbiota-host cometabolites (such as secondary bile acids) (12). Among different bacteria-derived metabolites, short-chain fatty acids (SCFAs) are a classic example of how bacteria-derived signaling molecules contribute to intestinal homeostasis and host health (13), particularly in herbivorous animals. SCFAs, including acetate, propionate and butyrate, serve as a major source of energy not only for ruminants but also for hindgut fermenters (14), and also exert a variety of distinct physiological responses in the host (15). Therefore, the differences observed between cecal and colon bacterial contents and their SCFA metabolites as well as their relations in the donkey hindgut should be taken into account to better understand microbe metabolism along the equine hindgut.

Donkeys are not only able to digest rough feeds, but also have strong disease resistance and adaptability (16, 17), which contribute to their specialized and enlarged hindgut. Previous studies have shown the key important roles of hindgut microbes and their metabolites in the regulation of metabolic function, intestinal pathogen resistance and immune homeostasis (18–20). The immune system plays an important role in governing host-microbe interactions in the intestine (19). Furthermore, the hindgut immune response is also easily activated as the hindgut contains a large amount of immune cells and some defense molecules, including immunoglobulin A (IgA) and antimicrobial peptides (AMPs) (21). In addition, in a study by van der Post et al. (22), mucus protein composition might be slightly different between the anatomical segments of the large intestine. There has not been a study performed on the regulation of the immune response along the donkey hindgut. On the other hand, the large intestine plays a critical role in the digestion and absorption of carbohydrates, including cellulose and undigested starch, in donkeys; thus, a deep understanding of the metabolic profiles (mainly lipid metabolism) along the donkey hindgut at the transcriptomic level is also important. Notably, it has been shown that there is close interplay between the gut microbiome and host gene expression in maintaining host health. For instance, in a recent paper published in *Science*, Bergstrom et al. demonstrated that the microbiota could induce the expression and production of Muc2 in the proximal colon, which then modulating the microbiota communities in the mouse colon (23). Due to physiological and anatomical structure differences, the spatial differences in dominant bacteria along the equine hindgut (24) suggest that these variations may influence the expression of genes in the different donkey hindgut segments. By providing the tissue samples from different areas of the hindgut (cecum and colon), it can perhaps provide insight into whether the host affects the microbial populations present within each compartment and how they interplay. To do this, an understanding of variation in molecular function at the transcriptional level along the hindgut is needed. We therefore compared the gene expression differences and explored the molecular regulation of lipid metabolism and immunity functions in the cecum and colon (four replicates each) using transcriptome analysis, which may provide insight into the intestinal molecular biology of donkeys.

The aim of this study was to investigate the difference along the donkey hindgut. First, we sought to evaluate and compare the microbial community and its function and the SCFAs in the cecum and colon of donkeys using metagenome sequencing and gas chromatography–mass spectrometry (GC-MS), respectively. Second, the interaction between the bacterial community and SCFAs was also performed to preliminarily explain the possible relationship between the bacterial community and its metabolites. To further our understanding of how commensal anaerobic bacteria regulate metabolism and immune functions in the hindgut, we assessed the differences in lipid metabolism and immune status along the donkey hindgut (cecum and colon) at the molecular level by whole transcriptomic analysis as well as the relationships among microbes, metabolites and host gene expression for the first time. The elucidation of the interaction between the gut microbiota and host molecular function related to lipid metabolism and the immune system, such as the difference along the donkey hindgut, could shed light on intestinal nutrition and immunity in donkeys.

## Materials and methods

### Animal selection, husbandry and sample collection

Six healthy male donkeys (age: 2.5 years; body weight: 232 ± 4 kg) were selected for this study; details were shown in **Supplementary Table 1**. All donkeys were raised under the same farming conditions in a Dezhou donkey breeding farm authorized by Shandong Province (Dezhou city, Shandong, China). Corn straw diets were fed ad libitum in addition to a commercial concentrate diet (Hekangyuan Group Co., Ltd., Shandong, China), and donkeys were fed twice daily at 07:00 and 19:00 (25). They were allowed free access to clean water at all times. Additionally, none of the donkeys had any previous history of GI disorder and were given antibiotics for at least 3 months before sampling. Moreover, the animal care protocol in this study followed commercial management practices and was approved by the Animal Welfare Committee of Liaocheng University (Permit No. DFG21010103-1).

**Figure 1:**
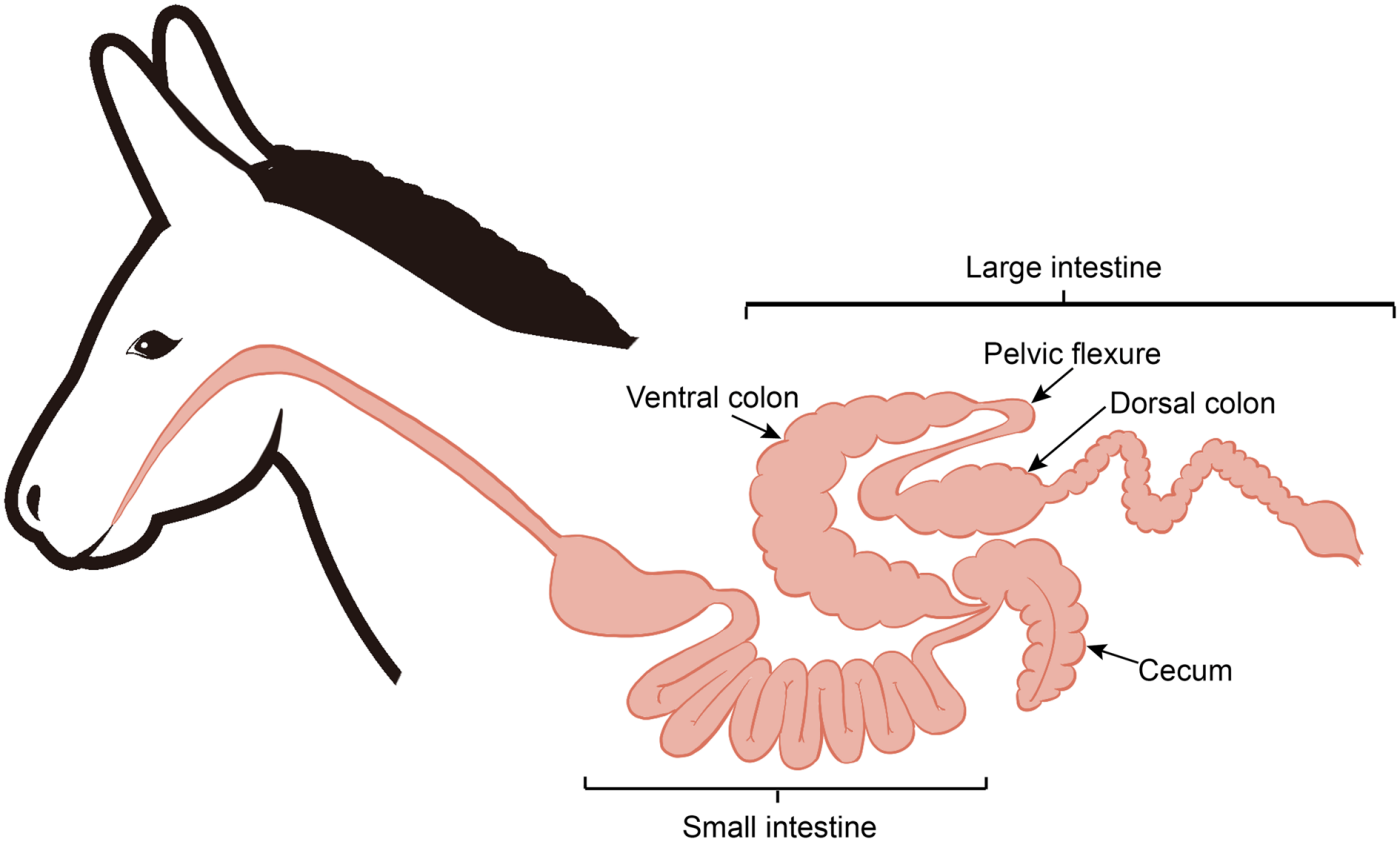
Diagram of the donkey intestinal tract.

After 12 hours of feed withdrawal, six donkeys were slaughtered for tissue collection, as described in our previous publication (25). Based on the anatomical structure differences along the donkey hindgut (**Figure 1**), the hindgut tissues (cecum, ventral colon, and dorsal colon) were separated, and the contents of each section were collected in this study. All samples were transferred to separate, sterilized 2 mL tubes and then frozen immediately in liquid nitrogen. Then, all frozen samples stored in dry ice were transported to the laboratory and stored at -80 °C for further analysis.

### Genomic DNA and genome sequencing

Total genomic DNA was extracted from 100 mg of frozen contents from the hindgut content (cecum, colon, ventral colon, and dorsal colon) of donkeys using an E.Z.N.A.^®^ Soil DNA Kit (Omega Bio-Tek, Norcross, GA, U.S.) according to the manufacturer’s instructions. DNA yield and quality were determined with a NanoDrop2000 (Thermo Scientific, Wilmington, USA). DNA fragments with an average size of approximately 400 bp were sequenced on an Illumina NovaSeq/HiSeq XTen instrument (Illumina Inc., San Diego, CA, USA) at Majorbio Bio-Pharm Technology Co., Ltd. (Shanghai, China) using NovaSeq Reagent Kits/HiSeq X Reagent Kits according to the manufacturer’s instructions (www.illumina.com).

### Metagenomics analyses

The data were analyzed on the Majorbio Cloud Platform (www.majorbio.com). The raw reads from metagenome sequencing were used to generate clean reads by removing adaptor sequences, trimming and removing low-quality reads (reads with N bases, shorter than 50 bp or with an average quality score < 20) using fastp (version 0.20.0) (26). The clean reads were mapped to the Dezhou donkey genome (https://www.ncbi.nlm.nih.gov/genome/7038?genome_assembly_id=1720012) using BWA (version 0.7.9a) to identify and remove the human host-originating reads with high scoring alignments. Then, these high-quality reads were assembled into contigs using MEGAHIT (version 1.1.2) (27), which utilizes SdBG. Contigs with lengths of 300 bp or more were selected as the final assembly result and used for further gene prediction, taxonomy, and functional annotation. Additionally, sequences were submitted to the NCBI Sequence Read Archive under accession number PRJNA860652.

We used Metagene to identify the open reading frames (ORFs) of contigs (http://metagene.cb.k.u-tokyo.ac.jp/). The predicted ORFs (≥100 bp) were retrieved and translated into amino acid sequences using the NCBI translation table. A nonredundant gene catalog was constructed using CD-HIT software (version 4.6.1) with 90% identity and 90% coverage (28). After quality control, reads were mapped to the nonredundant gene catalog with 95% identity using SOAPaligner (version 2.21), and gene abundance in each sample was evaluated *via* reads per kilobase of transcript per million mapped reads (RPKM). The taxonomic information was annotated based on the NCBI NR database using Diamond (version 0.8.35), as described previously (29). The Kyoto Encyclopedia of Genes and Genomes (KEGG) annotation was conducted by Diamond (version 0.8.35) against the KEGG database (version 94.2), while the Gene Ontology (GO) annotation was conducted by blast2go (http://www.blast2go.com). In addition, all these databases were carried out at an E-value cutoff of 1E-5 while ORF was annotated.

### Measurement of hindgut SCFAs

The concentrations of SCFAs in the hindgut content (six replicates per section) were measured using GC-MS. In brief, all samples (25 mg of hindgut content) were acidified with phosphoric acid, and SCFAs were extracted with 0.2 mL of butyl alcohol solvent containing 2-ethylbutyric acid (10 μg/mL) as an internal standard. After vortexing (10 s), ultrasonic extractions were performed for 10 min, followed by centrifugation at 13,000 rpm for 15 min at 4 °C. Then, the supernatant was carefully transferred to sample vials for analysis.

For GC-MS analysis, 1.0 μL of the sample was injected at a ratio of 10:1 at a constant flow rate (1 mL/min) on a HP-FFAP column measuring 30 m × 0.25 mm × 0.25 μm using a GC-MS spectrometer (8890B-5977B, Agilent). The GC column temperature was programmed to hold at 80 °C and rise to 120 °C at a rate of 40 °C per minute, then rise to 200 °C at a rate of 10 °C per minute, and finally hold at a temperature of 230 °C for 3 min. Data acquisition was conducted in full scan-selected ion monitoring mode. Compounds were identified and quantified using MassHunter software (v10.0.707.0, Agilent).

### RNA sequencing and analysis

Total RNA was extracted from 200 mg of hindgut tissue (cecum and dorsal colon) using TRIzol reagent (Invitrogen, USA) according to the instructions. RNA quality was verified using a 2100 Bioanalyzer (Agilent Technologies, Santa Clara, CA, USA) and a NanoDrop 2000 (Thermo Scientific, Wilmington, USA),and high-quality RNA samples (OD260/280 = 1.8~2.2, RIN≥8) were used to construct a sequencing library. The paired-end RNA-seq libraries were sequenced with an Illumina NovaSeq 6000 (2 × 150 bp read length) using 5 μg of total RNA. The RNA-seq data were analyzed with the free online Majorbio Cloud Platform (http://www.majorbio.com). Briefly, quantification of gene expression as transcript per million (TPM) values was carried out using the RSEM algorithm (Version 1.3.1) (30). The differentially expressed genes (DEGs) were identified and screened by DESeq2 based on |log 2FC| ≧ 1 and p value < 0.05 (31); subsequently, DEGs were used for functional enrichment analysis, including GO and KEGG pathway analysis. All data have been uploaded to the NCBI Sequence Read Archive (SRA) database (accession number: PRJNA860092). Finally, to validate the transcriptome results, ten mRNAs were randomly selected for qRT-PCR. The primer information for genes is listed in **Supplementary Table 2**.

### Statistical analyses

Taxonomic and transcriptome data for the cecum and colon were analyzed in the online Majorbio Cloud Platform, as were the functional profiles. For multiple group comparisons, the Kruskal-Wallis test was conducted followed by the Tukey-Kramer test. The Wilcoxon rank-sum test was used to detect the differences in metagenomic data between the two groups. Meanwhile, Student’s t test was used to analyze the differences in transcriptomics, RT-PCR data and SCFA levels between the two groups. Finally, Spearman’s correlation analysis was used to evaluate the correlations among the differentially enriched species abundance, SCFA concentrations, and DEGs. Means were considered significantly different when p < 0.05.

## Results

### Summary of metagenomics sequencing data

Twelve DNA samples extracted from the hindgut contents (cecum, ventral colon, and dorsal colon) of four donkeys were used for sequencing, generating metagenomic datasets totaling 162.26 Gb, with 91,033,216 ± 1,542,370 reads (mean ± SEM) per sample (**Supplementary Table 3**). Read numbers, quality filtration of raw reads, and the sequencing assemblies are summarized in **Supplementary Tables 3** and **Supplementary Table 4**. The represented Dezhou donkeys-derived reads were removed before analysis and are shown in **Supplementary Table 3**. After removing redundancies, a total of 794,403,348 genes were retained.

### Taxonomic composition of microbial communities along the hindgut of donkeys

**Figure 2:**
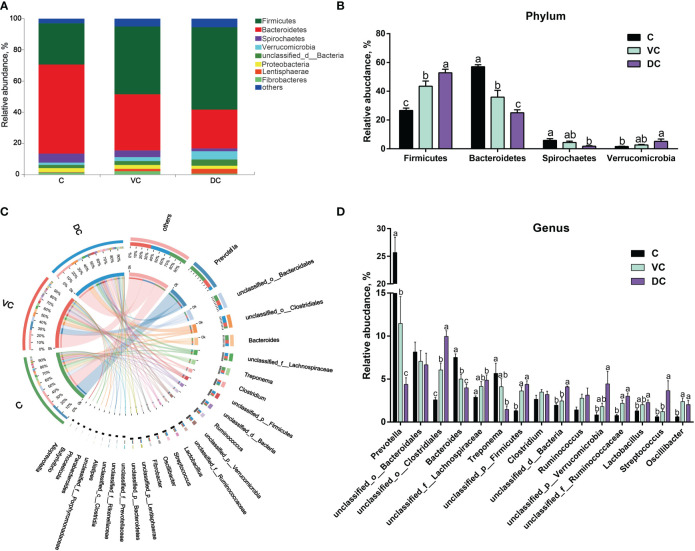
Taxonomic analysis along the hindgut microbiome in Dezhou donkeys. **(A)** Microbial community bar plot at the phylum level. **(B)** The relative abundance of the phyla Firmicutes, Bacteroidetes, Spirochaetes and Verrucomicrobia along the donkeys’ hindgut. **(C)** Circos diagram depicting the microbial community at the genus level in all samples. **(D)** The relative abundance of the top 15 genera along the donkeys’ hindgut. C, cecum; VC, ventral colon; DC, dorsal colon. Data indicate means ± SEM (n = 4), ^a,b,c^ Means with different letters are significantly different, p < 0.05.

Taxonomic profiling indicated that the cecal microbiota in donkeys was dominated by Bacteroidetes (57.07%), followed by Firmicutes (26.54%) and, to a lesser extent, Spirochaetes (5.85%), Proteobacteria (2.37%), unclassified_d:Bacteria (1.96%), Verrucomicrobia (1.59%) and Fibrobacteres (1.49%) (**Figure 2A**). However, the colon (ventral colon and dorsal colon) microbiota in donkeys was dominated by Firmicutes (43.55% and 52.92%, respectively), followed by Bacteroidetes (35.83% and 25.03%, respectively). As shown in **Figure 2B**, we observed that the cecum and colon differed significantly in the top 4 dominant phyla, including Firmicutes, Bacteroidetes, Spirochaetes and Verrucomicrobia.

At the genus level, a total of 5,257 genera were identified, of which 4,450 were shared by the three sections (**Supplementary Figure 1**). Overall, *Prevotella* was the most abundant genus in both the cecal and ventral colon microbiota, whereas *unclassified_o:Clostridiales* was more abundant in the dorsal colon microbiota (**Figures 2C, D**). Bacteroidetes contain two main genera, *Prevotella* and *Bacteroides*, and the enriched abundance of Bacteroidetes in the cecum was attributed to a higher abundance of *Prevotella* (**Figure 2D**).

**Figure 3:**
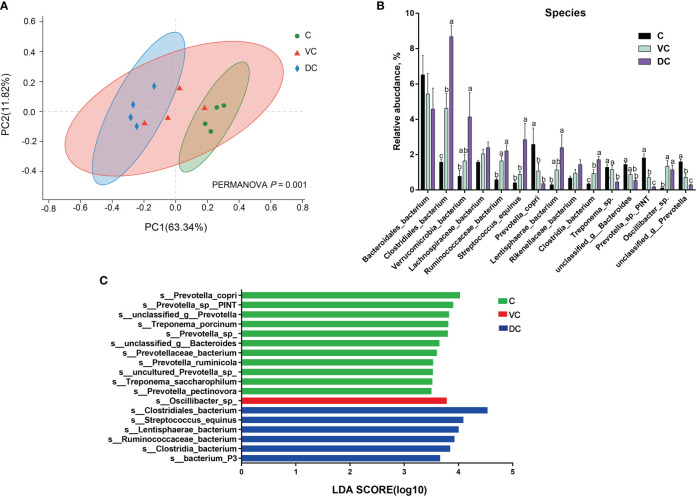
The distinct microbiota composition along the donkeys’ hindgut at the species level. **(A)** Principal coordinate analysis (PCoA) and PERMANOVA test (*P* = 0.001) based on Bray-Curtis distances of the microbiota along the hindgut in donkeys. **(B)** The relative abundance of the top 15 species along the donkeys’ hindgut. **(C)** Histogram of the LDA scores, showing the most differentially abundant taxa among the three sections (LDA score > 3.5, n=4). C, cecum; VC, ventral colon; DC, dorsal colon. Data indicate means ± SEM (n = 4), ^a,b,c^ Means with different letters are significantly different, p < 0.05.

At the species level, we found that there were substantial microbial differences between the cecal and dorsal colon digesta of donkeys based on the results of the PCoA and PERMANOVA tests (**Figure 3A**). After further analysis, our results revealed that the three most abundant species were *Bacteroidales_bacterium* (6.55%), *Prevotella_copri* (2.58%), and *Prevotella_sp._PINT* (1.81%) in the cecum; *Bacteroidales_bacterium* (5.40%), *Clostridiales_bacterium* (4.62%), and *Lachnospiraceae_bacterium* (2.04%) in the ventral colon; and *Clostridiales_bacterium* (8.67%), *Bacteroidales_bacterium* (4.58%), and *Verrucomicrobia_bacterium* (4.09%) in the dorsal colon (**Figure 3B**). We also used LEfSe analysis (LDA >3.5) to identify bacteria at the species level that accounted for the greatest differences in abundance among the three sections of the donkeys’ hindgut (**Figure 3C**). Eleven species (six of them belonging to the *Prevotella* genus) including *Prevotella_copri*, *Prevotella_sp:PINT*, *Prevotella_sp*, *Treponema_porcinum*, *unclassified_g:Prevotella*, *unclassified_g:Bacteroides*, *Prevotellaceae_bacterium*, *Prevotella_ruminicola*, *uncultured_Prevotella_sp*, *Treponema_saccharophilum*, and *Prevotella_pectinovora* were significantly enriched in the cecum, which indicated that the members from *Prevotella* can be considered the predominant the bacteria contributing to the difference among the three sections. We also observed that *Prevotella copri* was the most abundant *Prevotella* genus in the cecum (**Figure 3B**). Six species, namely, *Clostridiales_bacterium*, *Streptococcus_equinus*, *Lentisphaerae_bacterium*, *Ruminococcaceae_bacterium*, *Clostridia_bacterium*, and *bacterium_P3*, were significantly enriched in the dorsal colon, while only *Oscillibacter_sp* was mainly enriched in the ventral colon. Altogether, the taxonomic composition along the hindgut of donkeys at the phylum, genus, and species levels all revealed that there were significant differences in terms of dominant bacteria, especially between the cecum and dorsal colon.

### Functional profiles of microbes along the hindgut of donkeys

**Figure 4:**
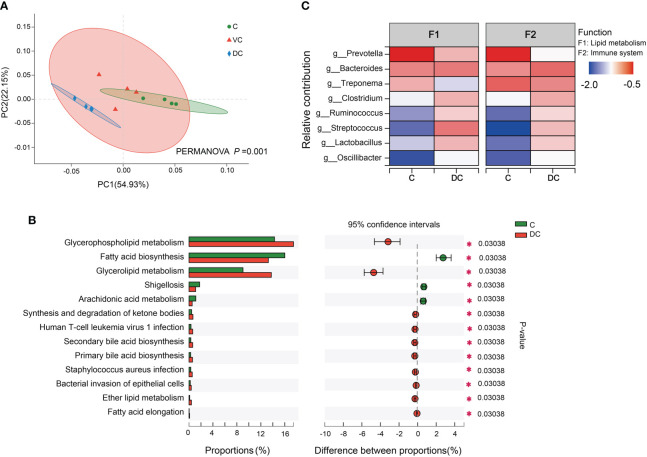
Functional profiles of the microbiota along the hind-gut in Dezhou donkeys. **(A)** Principal coordinate analysis (PCoA) and PERMANOVA test (*P* = 0.001) showing significant differences in Kyoto Encyclopedia of Genes and Genomes (KEGG) function in pathway level 3 of the C and DC groups. **(B)** Differential KEGG metabolic pathways were observed between the C and DC groups at level 3 (*p< 0.05). **(C)** Species contribution relationship of KEGG functions at the gene level in the C and DC groups. C, cecum; VC, ventral colon; DC, dorsal colon.

The data of the PCoA and PERMANOVA tests (p = 0.001) revealed significant differences in the microbial functions between the cecum and dorsal colon digesta (**Figure 4A**). In addition, we found that the difference in the taxonomic composition of the hindgut microbiota mainly existed between the cecum and dorsal colon. Then, we analyzed the functional profiles (including lipid metabolism and immune function) of the cecum and dorsal colon digesta based on KEGG annotation. After comparing the differences at level 3 between the two groups, the fatty acid biosynthesis pathway was more enriched in the cecum (16 ± 0.42%) than in the dorsal colon (13.25 ± 0.82%), while the glycerophospholipid and glycerolipid metabolism pathways were more enriched in the dorsal colon (17.45 ± 0.64%, 13.75 ± 1.23%) than in the cecum (14.28 ± 1.40%, 9.03 ± 0.36%; **Figure 4B**). Meanwhile, there was also a significant difference in disease infection between the two groups (**Figure 4B**), showing clear variation in the immune response along the donkey hindgut. Interestingly, antioxidant activity was higher in the cecum than in the dorsal colon based on GO annotation at level 2 (**Supplementary Figure 3**), which revealed that cecum microbes are more prone to activate the host antioxidant capacity compared to the dorsal colon. Moreover, the heatmap analysis of the species and their functional contribution revealed that Prevotella was the primary contributor supporting lipid metabolism and the immune system in the cecum, while the two functions mainly came from Streptococcus and Bacteroides in the dorsal colon (**Figure 4C**).

### Differentially expressed genes along the donkeys’ hindgut

**Figure 5:**
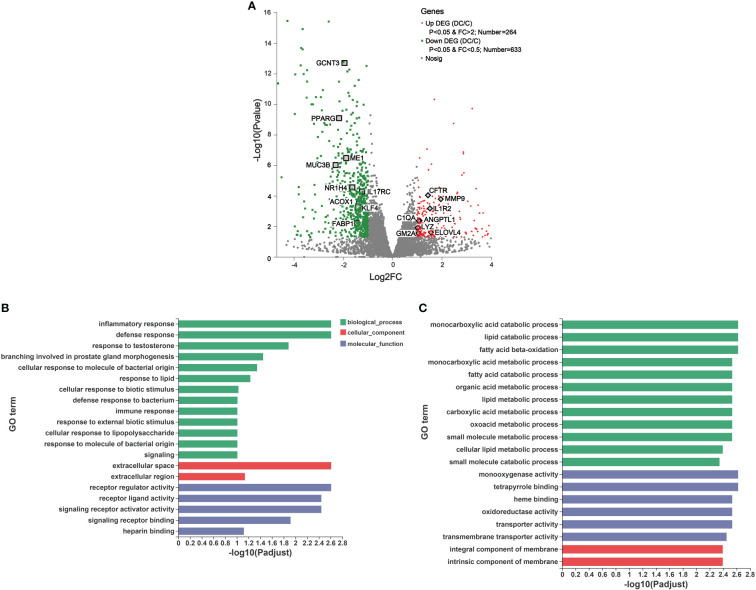
Transcriptomic analysis of the differentially expressed genes (DEGs) and their function in donkey hindgut tissues. **(A)** Volcano plot. **(B)** Gene Ontology (GO) functional enrichment analysis of the up regulated DEGs. **(C)** GO functional enrichment analysis of the down regulated DEGs.

To observe the differences in gene expression along the donkey hindgut, the cecum and colon tissue were collected to perform RNA sequencing. In total, 19, 770 genes were detected, and 897 DEGs (|log 2FC| ≧1 and p value < 0.05) were identified between the two sections (**Figure 5A** and **Supplementary Table 6A**). PCA also clearly revealed that the two groups were separated on PC1 (50.91%), indicating that there were visible differences in the gene expression between the cecum and dorsal colon (**Supplementary Figure 4**). We identified 633 DEGs (downregulated DEGs) that were enriched in the cecum, while 264 DEGs (upregulated DEGs) were enriched in the dorsal colon (**Figure 5A**). As mentioned above, we mainly focused on the differences in lipid metabolism and the immune system along the hindgut in this study. Regarding the lipid metabolism, we observed that the expression of some interesting genes involved in fatty acid biosynthesis, fatty acid oxidation and transport (such as *ME1*, *MBOAT1*, *ACOX1*, *ACOX2*, *LIPH*, and *PPARG*) was higher in the cecum than in the dorsal colon (**Figure 5A** **;** **Supplementary Table 6B**). We also observed that expression of genes in the most solute carrier families except the Na+/P+ exchanger (*SLC34A2*), was downregulated in the dorsal colon compared with the cecum; this includes genes encoding the mitochondrial fatty acid carriers (*SLC25A22*), monocarboxylate transporters (*SLC16A1* and *SLC16A5*), salt and water transporters (*SLC26A3*), nucleotide sugar transporters (*SLC35C1*), glucose transporters (*SLC50A1*), amino acid transporters (such as *SLC6A4* and *SLC6A8*), sodium- and chloride-dependent transporters (*SLC44A4*), and Na+/H+ exchangers (*SLC9A2*) (**Supplementary Table 6B**). Furthermore, genes mainly involved in mineral absorption, such as *S100G* and *VDR*, were more highly expressed in the cecum than in the dorsal colon (**Supplementary Table 6B**).

We also identified some interesting DEGs involved in immune and inflammatory responses, including *MUC3B*, *IL17RC*, *KLF4*, *IL1R2*, *C1QA*, *CFTR* and *MMP* (**Figure 5A**). Notably, there were significant differences in the expression of genes related to mucosal composition, including mucin-2-like, mucin-12-like, mucin-3B-like, mucin-5AC-like, *MUC13*, *MUC5AC* and *MUC6*, which indicated clear differences in mucosal immunity between the cecum and dorsal colon (**Supplementary Table 6B**). Additionally, some important genes involved in ROS production (e.g., *NOX1*, *NOXO1* and *DUOX2*) and the antioxidant system (such as *SELENOM*, *GSTK1*, glutathione S-transferase P and glutathione S-transferase P-like) were more highly expressed in the cecum than in the dorsal colon (**Supplementary Table 6B**), which is consistent with the findings of the microbiota function analysis based on the GO annotation at level 2 (**Supplementary Figure 3**). Furthermore, the expression of 10 DEGs related to lipid metabolism (*CPT1A*, *FABP1*, *LIPH*, *PPARD* and *PPARG*) and the immune system (*C1QA*, *LYZ*, *MUC6*, *GSTP* and *GCNT3*) was validated through RT-qPCR (**Supplementary Figure 5**), and showed an expression pattern consistent with those obtained by the RNA-Seq method.

To further understand the function of these DEGs, GO and KEGG enrichment analyses were performed (**Figures 5B, C** and **Supplementary Figure 6**). As evident from **Figure 5B**, in the molecular function (MF), cellular component (CC), and biological process (BP) categories, the upregulated DEGs were mainly concentrated on inflammatory responses, defense responses, response to lipids, and immune responses, on the extracellular space and extracellular region, and on receptor regulator activity, receptor ligand activity and signaling receptor activator activity. Furthermore, KEGG terms of these DEGs indicated that they were enriched in human diseases, organismal systems and metabolism, including *Staphylococcus aureus* infection (e.g., *C1QA* and cathelin), the Toll-like receptor signaling pathway (e.g., *CXCL8* and *CXCL9*) and IL-17 signaling pathway (e.g., *MMP1*, *MMP9* and *CXCL8*), and drug metabolism-cytochrome P450a and arachidonic acid metabolism (e.g., *HPGDS* and *PLA2G4D*), respectively (**Supplementary Figure 6A**). On the other hand, the top 20 GO analyses revealed that the downregulated DEGs were mainly involved in biological processes, including lipid catabolic processes, fatty acid beta oxidation, fatty acid catabolic processes and cellular lipid metabolic processes, as well as cellular components, such as monooxygenase activity and oxidoreductase activity (**Figure 5C**). Moreover, it is not surprising that based on the KEGG pathway analysis, downregulated DEGs were mainly enriched in drug metabolism, fatty acid degradation, mineral absorption and the PPAR signaling pathway (**Supplementary Figure 6B**).

### Correlation and association analysis between differentially expressed genes and differential species

**Figure 6:**
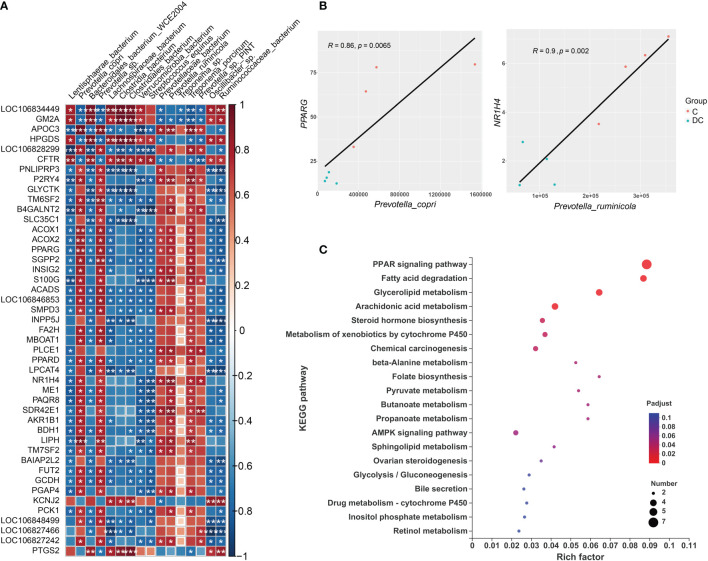
Interactions between hindgut microbes and genes associated with lipid metabolism. **(A)** Correlation plot depicting the correlation between the gut microbial population at the species level based on the LEfSe results (in a total of 16 species) and the expression levels of lipid metabolism genes. The color of the squares shows the magnitude of the correlation (the absolute value of the correction coefficient denoted by r was set to 0.7 or greater), and asterisks indicate the significance of the correlation (*p< 0.05; **p< 0.01; ***p< 0.001). **(B)** Scatterplots illustrating the pattern of grouping by C (red) and DC (blue) samples in two representative microbe-gene correlations, where the strength of the correlation (r value) and significance (p value) are shown at the top of each plot. **(C)** The Kyoto Encyclopedia of Genes and Genomes (KEGG) functions of the DEGs associated with differentially enriched species.

**Figure 7:**
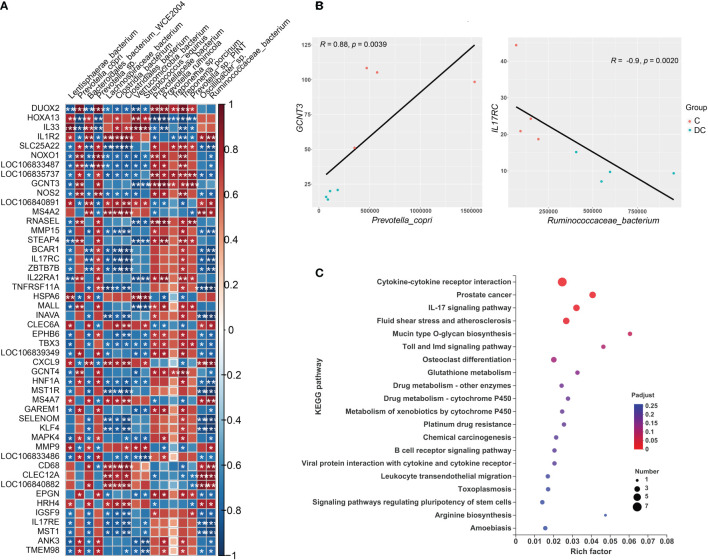
Interactions between hindgut microbes and genes related to the immune system. **(A)** Correlation plot depicting the correlation between the gut microbial community at the species level based on the LEfSe results (in a total of 16 species) and the expression levels of immune system genes. The color of the squares shows the magnitude of the correlation (the absolute value of the correction coefficient denoted by r was set to 0.7 or greater), and asterisks represent the significance of the correlation (*p< 0.05; **p< 0.01; ***p< 0.001). **(B)** Scatterplots illustrating the pattern of grouping by C (red) and DC (blue) samples in two representative microbe-gene correlations, where the strength of the correlation (r value) and significance (p value) are shown at the top of each plot. **(C)** The Kyoto Encyclopedia of Genes and Genomes (KEGG) functions of the DEGs associated with differentially enriched species.

The gut microbiota plays an important role in host metabolism and the immune system. This study performed correlation analyses of different gene expression levels and differentially enriched species based on the LEfSe results (**Supplementary Figure 2**). Using Spearman’s rank correlation, a total of 16 species were related to 189 DEGs (**Supplementary Tables 6C, D**), where the absolute value of the correction coefficient denoted by r was set to 0.7 or greater. We mainly focused on the DEGs related to lipid metabolism and the immune system, and all the correlations between them and taxon abundances are shown in **Figure 6A** and **Figure 7A**. Our results revealed complex correlations between the hindgut microbiota and expression of host genes involved in lipid metabolism and the immune system (**Supplementary Tables 6E, F**). With respect to lipid metabolism, most microbial species enriched in the cecum, especially *Prevotella copri*, *Prevotella sp* and *Treponema porcinum*, were positively correlated with some important genes mainly involved in fatty acid biosynthesis (*ME1* and *MBOAT1*), fatty acid oxidation(*ACOX1*, *ACOX2* and *LIPH*), and fatty acid transport (*APOC3*) (**Figure 6A**). However, most microbial species enriched in the dorsal colon, especially *Streptococcus equinus* and *Ruminococcaceae bacterium*, were negatively correlated with the expression of these genes (**Figure 6A**). Additionally, **Figure 6B** depicts the pairwise correlations between the microbial taxa and lipid metabolism gene expression, both of which were previously reported to be involved in host lipid metabolism and thus may be of interest. For instance, the transcription factors *PPARG* and *NR1H4* (also called FXR), as important regulators of lipid metabolism, are positively correlated with *Prevotella copri* (Spearman rho = 0.86, p value = 0.0065) and *Prevotella ruminicola* (Spearman rho = 0.9, p value = 0.002), respectively, which affect host fat deposition. To further evaluate the functional profiles of the differentially enriched species related to lipid metabolism DEGs, we also annotated the KEGG pathways of these DEGs (**Figure 6C**). These DEGs were mainly located in the PPAR signaling pathway, fatty acid degradation, glycerolipid metabolism, arachidonic acid metabolism, and the AMPK signaling pathway.

Regarding the relationship of microbial taxa and immune response gene expression, we found that there were largely complex positive/negative gene-taxa correlations. For instance, most *Prevotella* enriched in the cecum were positively correlated with the expression of genes involved in reactive oxygen species (ROS) production (such as *NOXO1* and *DUOX2*) and antioxidant activity (e.g., *GST-P*) and negatively correlated with TH2 inducers (such as *IL33* and *IL1R2*). However, most species enriched in the dorsal colon, especially *Streptococcus equinus* and *Verrucomicrobia bacterium*, were significantly positively correlated with the expression of some genes involved in the oxidative response (e.g., *HOXA13* and *HSPA6*), TH2 induction (e.g., *IL33*) and innate immunity (e.g., *LILR* and *MMP9*) and negatively correlated with the expression of some genes involved in ROS production (*DUOX1* and *NOXO1*) and the mucosal barrier (*GCNT3* and *MUC3B*-like). **Figure 7B** also displays the two interesting pairwise correlations between the microbial taxa and immune system gene expression and shows a significant positive correlation between *GCNT3* and *Prevotella copri* (Spearman rho = 0.88, p value = 0.0039), while *IL17RC* was negatively correlated with *Ruminococcaceae_bacterium* (Spearman rho = -0.9, p value = 0.0020). Additionally, we also annotated the KEGG pathways of immune response DEGs related to these species and revealed that it mainly focused on cytokine-cytokine receptor interaction, prostate cancer, IL17 signaling pathway, fluid shear stress and atherosclerosis, mucin type O glycan biosynthesis, Toll and Imd signaling pathways, osteoclast differentiation, and glutathione metabolism (**Figure 7C**).

**Figure 8:**
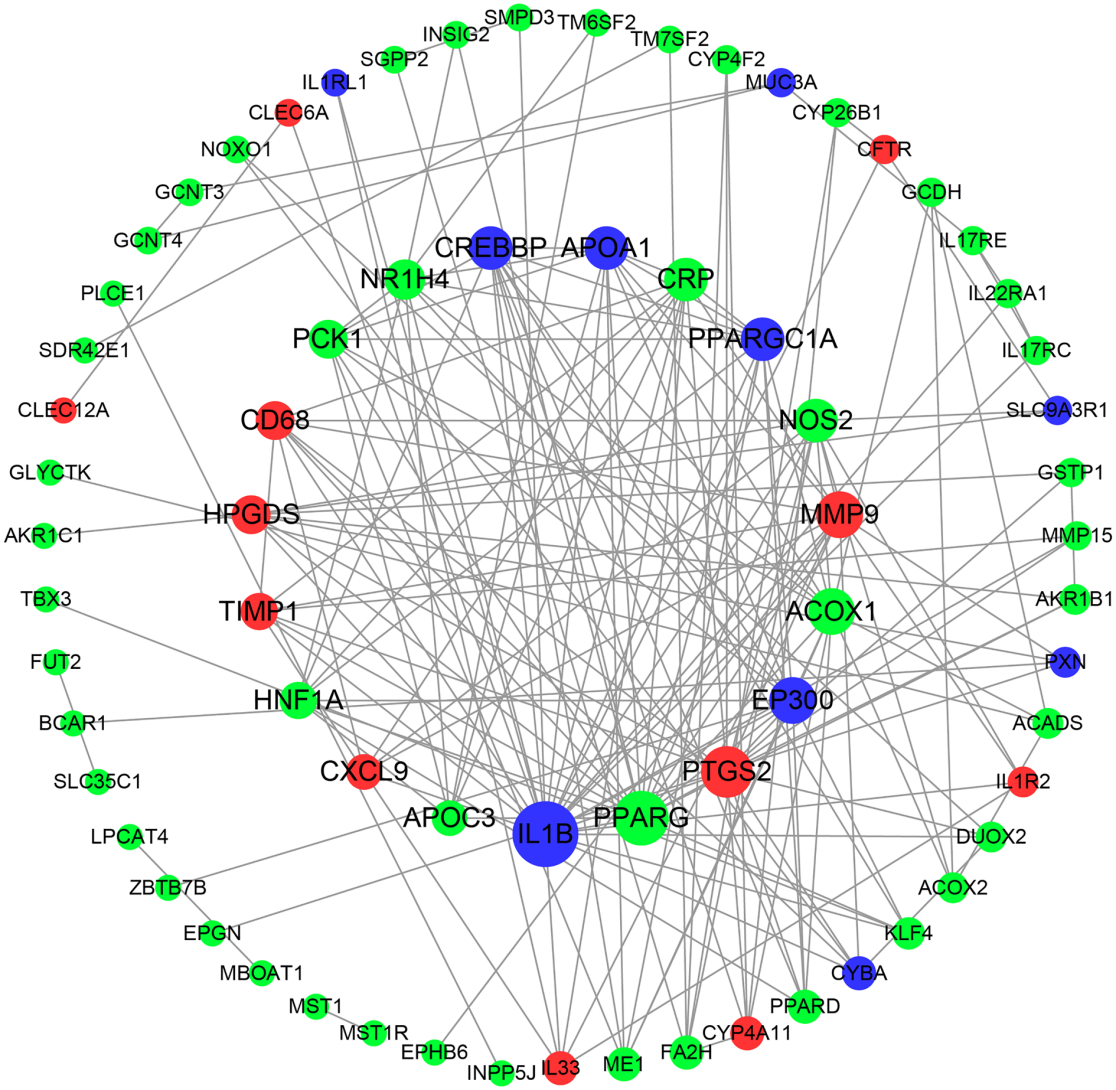
Protein-protein interaction (PPI) network of DEGs correlated with species. Red nodes indicate up-regulated genes, and green represents down-regulated genes. Node size indicates the degree; a bigger node indicates a higher degree.

Based on the close relationship between lipid metabolism and the immune response, the 93 DEGs (combining those associated with lipid metabolism and the immune response) related to these species were input into the STRING database, and the PPI network was constructed and visualized using Cytoscape software (**Figure 8**). We identified the top 10 hub genes, which were *PPARG*, *PTGS2*, *ACOX1*, *MMP9*, *NOS2*, *CRP*, *NR1H4*, *PCK1*, *HPGDS*, and *CD68*. Considering the role of *PPAR* in metabolism and inflammation, the PPAR pathway is likely responsible, at least in part, for the role of the hindgut microbiota in intestinal fatty metabolism and immune regulation in donkeys.

### Correlation and association analysis of SCFA with differentially enriched species and differentially expressed genes

First, we measured the SCFA concentrations in the cecum and dorsal colon (n = 6 per region) by targeted metabolomics in donkeys. Apart from propionate, the concentrations of acetate, isobutyrate, valerate and isovalerate were all lower in the cecum than in the dorsal colon (p < 0.05, **Supplementary Table 5**). However, the concentrations of butyrate and total SCFAs did not differ significantly among the hindgut sections (p > 0.05).

Considering the possible relationship between SCFA and the gut microbiota, as well as the host gene expression, the present study further performed the Spearman’s rank correlation of differential SCFA and differentially enriched microbial species (**Supplementary Figure 7**) and differentially expressed genes (the absolute value of r was set to 0.8 or greater; **Supplementary Figure 8**). Our data suggested that there were significant correlations between the gut microbiota and SCFAs (**Supplementary Table 6G**). For instance, the microbial species, including four *Prevotella* species (*Prevotella_copri*, *Prevotella_sp*, *Prevotellaceae_bacterium* and *Prevotella_ruminicola*) and two *Treponema* species (*Treponema_sp* and *Treponema_porcinum*), were all positively associated with propionate, while species enriched in the dorsal colon (except *Oscillibacter_sp*) showed a negative association with propionate. However, species enriched in the cecum were all negatively related to acetate, and only species enriched in the dorsal colon, including *Lentisphaerae_bacterium*, *Verrucomicrobia_bacterium* and *Streptococcus_equinus* were positively associated with acetate. Regarding BCFAs (isobutyrate, isovalerate and valerate), there were complex and discordant relationship with the differentially enriched species. Among the DEGs, *RHOF*, LOC123284254 (also called mitochondrial translational initiation factor 2; *MTIF2*), *NKX3-1*, *GATA4* and *GCNT3* were positively correlated with propionate, and negatively correlated with acetate isobutyrate, valerate, and isovalerate (**Supplementary Table 6H**).

## Discussion

The intestinal microbiota and its metabolites (such as SCFAs), as important environmental regulatory factors, play a key role in modulating host metabolism and maintaining intestinal homeostasis (12), thereby affecting animal health and many disease states. Thus, understanding what/how contributes to a healthy, homeostatic relationship between the gut microbiota and host immune system and metabolism is of critical importance. More importantly, comprehensive knowledge of the roles played by the hindgut microbiota community and its metabolites as well as their interaction in host metabolism and the immune state may provide novel insights for improving anima metrics (such as product quality) and welfare in donkeys, as a classical hindgut fermenter. However, no such knowledge in donkeys is currently available. To fill this gap, we herein performed comparisons of the microbiota community, its main metabolites (SCFAs) and the expression of genes involved in lipid metabolism and the immune system, as well as their relationships between the cecum and colon in donkeys.

Similar to those in other mammals (23, 24, 32), the microbial diversity and composition of the donkey hindgut also varied significantly between the intestinal segments. Our study is the first to reveal distinct differences in the microbiota along the donkey hindgut using a metagenomics method. Firmicutes and Bacteroidetes were the most abundant phyla in the donkey hindgut in the current study, which is consistent with previous findings (1, 32, 33). Our findings demonstrated that Firmicutes (mainly including the families Ruminococcaceae and Lachnospiraceae) gradually increased along the donkey hindgut, yet Bacteroides (mainly including the families Prevotellaceae and Bacteroidaceae) showed the opposite trend. Similarly, in pigs, Firmicutes abundance in the colon was higher than that in the cecum, while Bacteroidaceae abundance in the colon was lower than that in the cecum (34). However, one previous study reported that the proximal large intestine is rich in Lachnospiraceae, while the distal large intestine is rich in Prevotellaceae in the horses (35). This discrepancy in findings is probably due to differences in many aspects, including various host factors, diet and environments. Combining the analysis at other levels (genus and species), we found clear differences in the composition profiles of the hindgut microbiome among the cecum and colon sites, especially between the cecum and dorsal colon sites, which revealed specific microbes with a preference for colonizing certain regions. As previously shown, the differential microbial composition across the intestine tract can be governed by multiple factors, including anatomical structure (24, 35), pH, oxygen levels, nutrient profiles, and the rate of nutrient flow. For instance, the pelvic flexure might be a key anatomical landmark that results in distinct microbial communities in the equine hindgut (24).

The characteristics and functional profile of some core bacteria detected in our study have been reported in previous research. For instance, six species belonging to the *Prevotella* genus that were abundant in the cecum in the current study have been widely accepted as having an ability to increase the capacity of the gut microbiota to ferment complex indigestible carbohydrates from the diet (36). *Prevotella_copri* isolated from pigs fed commercial formula diets can increase host fat accumulation and lead to an inflammatory response (37). Recently, the species belonging to the *Prevotella* genus (especially *Prevotella_copri*) have drawn broad interest partly due to their controversial roles (including metabolism and immune response) as important intestinal bacteria (38). It is also supported that *Prevotella* may be a potential probiotic in healthy adult animals (39), although its exact functional role (harmful and beneficial effects) remains to be elucidated in future work. *Treponema*, a member of the Spirochaetes phylum closely associated with cellulose digestion and utilization (40) and hemicellulose degradation (41), is also more abundant in the cecum. Furthermore, in pigs, *Treponema* is the largest contributor to high feed efficiency (42). It is possible that the higher abundance of *Prevotella* and *Treponema* in the cecum hints at the presence of a bacterial community using carboxymethylcellulose, xylan and xylose to produce more SCFAs (43), which may be why the level of propionate was higher in the cecum than in the dorsal colon. Accordingly, we also observed that the propionate content was positively correlated with most species belonging to *Prevotella* and *Treponema*. However, diverse species of major gut commensals, such as *Clostridium* (cellulose-degrading bacterium), *Streptococcus* (starch-utilizing bacterium), *unclassified_f:Lachnospiraceae* (complex carbohydrate-degrading bacterium), *Ruminococcus* (cellulose-degrading bacterium) and *Lentisphaera* (mucin-degrading bacterium), were identified and mainly enriched in the dorsal colon in this study. In line with a previous research, Liu et al. also demonstrated that the relative abundance of *Streptococcus* was higher in the colon than in the cecum (1). These findings suggested that the diversity of microbiota communities in the colon is possibly driven by complex substrates, including cellulose and undigested starch, as well as mucus derived from the proximal large intestine (including the cecum), which interplay and maintain intestinal homeostasis. For example, *Clostridium* could exert multiple salutary effects on intestinal barrier homeostasis by producing SCFAs (especially butyrate) (44). Interestingly, although there was no change in butyrate, isobutyrate and isovalerate (BCFA) contents were significantly higher in the dorsal colon than in the cecum, which indicates ongoing higher protein fermentation activity in the dorsal colon than in the cecum (45). Meanwhile, previous research by Davila et al. (46) and Ma et al. (47) showed that the proteolytic activity was mainly related to some genera, including *Bacteroides, Streptococcus, Lactobacillus*, *Clostridium*, and *Prevotella*, which were identified in this study in the large intestine of monogastric animals. In ruminants, BCFAs have been reported to promote the growth of some ruminal cellulolytic bacteria, such as *Ruminococcus* (48). Of note, the abundance of the species *Bacterium_P3* (belonging to the *Bacteroides* genus) was observed to be much higher in the colon of the donkeys than in the cecum. *Bacterium_P3* is a newly discovered species, and a recent study found that it has potentially high metabolic activity in the cattle rumen based on RNA data (49). This is the first time that *Bacterium_P3* has been found in the hindgut digesta of donkeys, and its exact function needs further study. Taken together, these findings suggest that the donkey hindgut selectively filter specific microbial members to function as residents in different regions, thereby benefiting the host. However, considering that many of the dominant bacterial genera and species belong to unclassified bacteria in the current study, indicating that knowledge about the hindgut of donkeys is still in its early stages, a deeper understanding and more basic research are needed.

The DEGs were analyzed to understand the distinct genomic differences between the cecal and colon sites. In total, 633 DEGs were highly expressed in the cecum, and 264 DEGs were significantly highly expressed in the dorsal colon. Because a primary objective was to characterize the differences in lipid metabolism and the immune system along the donkey hindgut, we focused on the enriched lipid and immune-related genes. We further investigated whether the transcriptional profiles related to lipid metabolism from different compartments varied along the donkey hindgut. These data indicate that the dorsal colon of donkeys may be weaker in lipid metabolism than the cecum, characterized by lower fatty acid biosynthesis (such as *ME1*, *MBOAT1* and *MOGAT1*) and fatty acid oxidation (such as *ACOX1*, *ACOX2* and *LIPH*), and weaker fatty acid transport (such as *APOC3* and *FABP1*). GO and KEGG enrichment analyses revealed that the fatty acid degradation and PPAR signaling pathways were mainly enriched in the cecum, while the arachidonic acid metabolism was enriched in the dorsal colon. PPARs play a vital role in regulating some aspects of lipid metabolism and energy balance (50), suggesting that they might contribute to the lipid metabolic difference between the cecum and dorsal colon. Correspondingly, the results of microbiota functional profiles also noted that the fatty acid biosynthesis pathway is more active in the cecum than in the colon. A similar comparative analysis was performed in sheep and showed that the cecum had a higher lipid absorption capacity than the proximal colon (51). In this study, we also found that the expression of most solute carrier family genes related to mitochondrial metabolism and energy metabolism was downregulated in the dorsal colon compared with the cecum. Moreover, the mineral absorption pathway was also mainly enriched in the cecum. Collectively, our data suggested that there are significant differences in the function of nutrient absorption between the cecum and dorsal colon in donkeys, especially in regard to lipid metabolism. This finding in fact has been confirmed by a previous study, which reported that the colon was weaker than the cecum in terms of its fermentation and absorption functions (52). These distinct differences may be due to environmental pressures, including lower water and nutrient levels (53) and clear differences in the microbiota.

The key role of microbial colonization in maintaining intestinal immune homeostasis is well recognized (54); in other words, some specific bacterial species in the gut can fine-tune gut immune responses, including T-cell and B-cell activation (32). However, information regarding its contributions and relationship with the intestinal immune system in donkeys’ hindgut is lacking. In doing so, we also highlighted the previously unappreciated spatiality difference in the immune response along the donkey hindgut. There is marked regional variation in immune status along the donkey hindgut. Of note, 98 interesting DEGs were screened based on the GO and KEGG annotation between the two regions. Overall, according to gene functional classification and screening, we observed a unique immune niche existing in different hindgut regions in healthy donkeys (**Supplementary Table 6C**). In vertebrates, the immune system is a complex multilayered system for defending the body against external and internal threats and maintaining host health. In regard to the innate immune system, the cecum was mainly enriched in the activation of innate immune cells, including macrophages (*MST1* and *MST1R*) and neutrophils (*SECTM1*), as well as innate immunity activator (*INAVA*) and matrix metalloproteinase proteins (*MMP15* and *MMP28*), yet the dorsal colon was mainly shown to have higher expression of macrophages (M1; CD68), complement system (*C1QA*) and matrix metalloproteinase proteins (*MMP1* and *MMP9*). We also noticed significant regional differences in the abundance of both T and B-cell (adaptive immunity context) lineage-specific genes. For instance, the gene abundance of some receptors related to IL17 (IL-17RC and *IL-17RE*) and IL22 (*IL22RA1*) was higher in the cecum than in the dorsal colon, which may be due to the higher proportion of Th17 cells in the cecal site. A decrease in the abundance of Th17 cells has been observed from the cecum to the sigmoid colon in mice (32). Conversely, the dorsal colon showed a higher abundance of IL1 receptors (*IL1R2* and *ILRN*) and IL33 expression as Th2 cell inducers, which meant that the dorsal colon may be dominated by Th2 cells. These data indicated differences in both the innate and adaptive immunity systems between the cecum and dorsal colon. Interestingly, the expression of genes related to mucin protein was mainly enriched in the cecum rather than in the dorsal colon, which was probably due to the greater abundance of mucus-associated bacteria in the cecum than in the dorsal colon (55). Our observations support a previous study showing that luminal mucus in the mice colon is mainly derived from goblet cells in the proximal colon (including the cecum) (23). Further research is also needed to confirm how mucus-associated bacteria work in this context and the changes at the protein level. Additionally, there are significant differences in the antioxidant level between the cecum and dorsal colon, indicating that the cecum may be more prone to oxidative stress and activation of the antioxidant system than the dorsal colon in donkeys. This finding was confirmed by the increased expression of some genes related to oxidative (such as *NOXO1* and *DUOX2*) and antioxidant (such as selenom and glutathione S-transferase, including *GSTK1*, glutathione S-transferase P and glutathione S-transferase P-like) systems, which was supported by the previous finding by Aviello and Knaus (56) that NOX/DUOX NADPH are the only enzymes that contribute to ROS production; they are multifaceted regulators with respect to intestinal epithelial homeostasis. It has also been reported that the colon has a lower antioxidant capacity than other intestine segments (57). However, the expression of genes related to antimicrobial peptides (such as cathelin and *LYZ*) was higher in the dorsal colon than in the cecum, which indicates variations in the level of protection needed along the donkey hindgut. For instance, cathelin-related antimicrobial peptide (*CRAMP*) plays an important role in supporting normal colon mucosal homeostasis (58). Collectively, these data underscore the specific immune functional profiles of these two hindgut regions. This distribution of genes related to lipid metabolism and immune response as well as antioxidant ability is consistent with the spatial composition and functional variation of the specific bacterial species, hinting at a close relationship between them.

Finally, to observe the relationship between the intestine microbiome and intestinal gene expression, we further performed Spearman’s correlation analysis for the differentially enriched species based on the LEfSe results and the DEGs of interest. As expected, we found that some gut microbial species that were highly enriched in the cecum, including *Prevotella_copri*, *Prevotella_sp*, *Prevotella_ruminicola* and *Treponema_porcinum*, were positively associated with lipid metabolism-related genes, including *ME1*, *MBOAT1*, *ACOX1*, *ACOX2* and *LIPH*. However, some species that were highly abundant in the dorsal colon, mainly *Verrucomicrobia_bacterium* and *Streptococcus_equinus*, were negatively correlated with the genes mentioned above. These results suggest that the expression of lipid metabolism (especially the fatty acid biosynthesis and fatty oxidative pathway) genes in donkeys may be associated with the prevalence of *Prevotella* (especially *Prevotella_copri*) and Spirochaetes in the cecum. Combining the references mentioned above, we raise the question of whether *Prevotella* could be a candidate for improving the intramuscular fat (IMF) of donkeys’ muscle to solve the bottleneck of lower IMF in the donkey industry. Future prospective research is needed. In addition to lipid metabolism, we also focused on the DEGs related to immune status, and we observed that most differentially enriched species were related to these genes. Several microbes identified in our study have previously been shown to modulate the immune response, such as *Prevotella copri* (59), Clostridiales and Verrucomicrobia (60). Interestingly, some species belonging to the *Prevotella* were positively correlated with some receptors related to IL17 and IL22 (Th17 cytokines), which is consistent with a significant capacity of *Prevotella* bacteria to drive the Th17-mediated mucosal inflammatory response (61). With respect to the negative association, the expression of the cytokine IL17 correlated negatively with the increased abundance of *unclassified Ruminococcaceae* (Clostridiales order) (60), which is similar to our finding. In addition to their immunologic effects, Th17 cells also serve to maintain gut barrier integrity in a healthy state (62). Moreover, *Prevotella* was positively correlated with expression of the antioxidant gene glutathione S-transferase P (LOC106833486) and intestinal mucous barrier genes (such as *GCNT3*, *GCNT4* and mucin 3B-like), suggesting that *Prevotella* could be an ideal candidate for regulating the immune response and maintaining the intestinal homeostasis in healthy donkeys. Enriched species in the cecum were also positively correlated with the expression of T-cell differentiation genes, such as *ZBTB7B*, *MALL* and *TMEM98*. For example, the secreted form of *TMEM98* can promote the differentiation of T helper 1 cells (Th1) (63). However, most species enriched in the dorsal colon were positively correlated with the expression of the IL33 gene, a Th2-type immune inducer (64), showing different cytokine profiles and distinct effector functions between the dorsal colon and cecum. In addition, some enriched species in the dorsal colon were also positively correlated with the expression of the leukocyte immunoglobulin-like receptor (*LILR*) family (LOC106840891 and LOC106840882), innate immune receptor (*CLEC6A*; also called Dectin-2), inflammatory chemokine ligands (such as *CXCL9*) and matrix metalloproteinase proteins (*MMP9*), suggesting that these species, as gut commensal bacteria, may also be involved in the activation of the intestinal innate immune response and thereby control the growth of enteric pathogens. In return, the immune system also plays a critical role in maintaining the symbiotic relationship of highly rich and diverse microbes, especially in the large intestine of the host (65). In other words, host genetics are also a critical factor affecting gut microbiome composition. Likewise, the protective role against intestinal inflammation was mainly driven by the abundance of the Lachnospiraceae family in Dectin-1/Dectin-2 KO mice models (66). Interestingly, we observed that the expression of genes related to ROS production (*NOXO1* and *DUOX2*) was significantly positively correlated with most of the enriched species in the cecum but was significantly negatively associated with many species enriched in the dorsal colon. Although NADPH oxidases such as *NOXO1* and *DUOX2* are intimately involved in crosstalk with the intestinal microbiota and vice versa (56), the relationship of differentially enriched species identified in our study and the exact regulatory mechanisms regarding this context are still unclear and need further research. Keeping in mind the effect of microbiota-derived metabolites (e.g., SCFAs) on the host, we found that the gut microbiota may affect gut function and balance *via* SCFAs; for instance, propionate was positively correlated with the expression of the *GCNT3* gene. We also found that the gene *NR1H4* (also called *FXR*), which is related to bile acids, was associated with some species identified in the hindgut, which indicates that the secondary bile acids are also metabolites involved in the effect of the hindgut microbiota on host function in donkeys. Notably, we observed that *PPARγ* abundance was positively or negatively correlated with the most differentially enriched species identified in our study as well as a core hug gene (based on the PPI analysis), suggesting that the *PPAR* pathway is responsible, at least in part, for the role of the hindgut microbiota in intestinal fatty metabolism and immune regulation in donkeys. Li et al. recently proposed a molecular mechanism for barley leaf protection against colitis by the gut microbiota-inosine-A2AR/PPARγ axis (67). To the best of our knowledge, our work is the first to focus on a comprehensive association analysis of the intestinal microbiome and intestinal gene expression along the donkey hindgut, especially with respect to lipid metabolism and immune status.

In conclusion, our work showed the compositional and functional differences along the hindgut microbiota as well as the distinct changes in gene expression related to lipid metabolism and immune response between the cecum and colon. Additionally, our correlation analyses of the hindgut microbiota and neighboring genes highlight the significance of microbiota as an important environmental signal in maintaining regional normal host health, and suggest that the PPAR pathway might be responsible, at least in part, for the role of the hindgut microbiota on donkeys’ gut homeostasis. However, the current study was limited to small numbers of bacteria, and the role of a large and complex microbial community in the intestinal metabolism and barrier homeostasis requires further exploration. Although further in-depth research is still needed, the correlations observed in this study provide novel information for studies on the interaction of the microbiota and gut function.

## Data availability statement

The original contributions presented in the study are publicly available. The data presented in the study are deposited in the National Center for Biotechnology Information Sequence Read Archive repository, accession number PRJNA860652 and PRJNA860092.

## Ethics statement

The animal study was reviewed and approved by Animal Welfare Committee of Liaocheng University.

## Author contributions

YL conceived the study. YL and QM drafted the manuscript. YL, QM, XS and GL performed the experiments. GL and CW supervised the work and reviewed the manuscript. All authors contributed to the manuscript and approved the submitted version.

## Funding

This work was supported by the National Natural Science Foundation of China (grant no. 32102564), the Open Project of Shandong Collaborative Innovation Center for Donkey Industry Technology (grant no. 3193308) and the Open Project of Liaocheng University Animal Husbandry Discipline (grant no. 319312101-11 and 319312101-13).

## Conflict of interest

The authors declare that the research was conducted in the absence of any commercial or financial relationships that could be construed as a potential conflict of interest.

## Publisher’s note

All claims expressed in this article are solely those of the authors and do not necessarily represent those of their affiliated organizations, or those of the publisher, the editors and the reviewers. Any product that may be evaluated in this article, or claim that may be made by its manufacturer, is not guaranteed or endorsed by the publisher.
