# Supplementary material for: Integrated multi-omics reveals novel microbe-host lipid metabolism and immune interactions in the donkey hindgut
Source: Front Immunol. 2022 Nov 18;13:1003247. doi: 10.3389/fimmu.2022.1003247 (PMC9716284; doi:10.3389/fimmu.2022.1003247)
Supplement: Supplementary file 1 [file DataSheet_1.doc]

Supplementary Materials

# Supplementary Figures and Tables

## Supplementary Figures


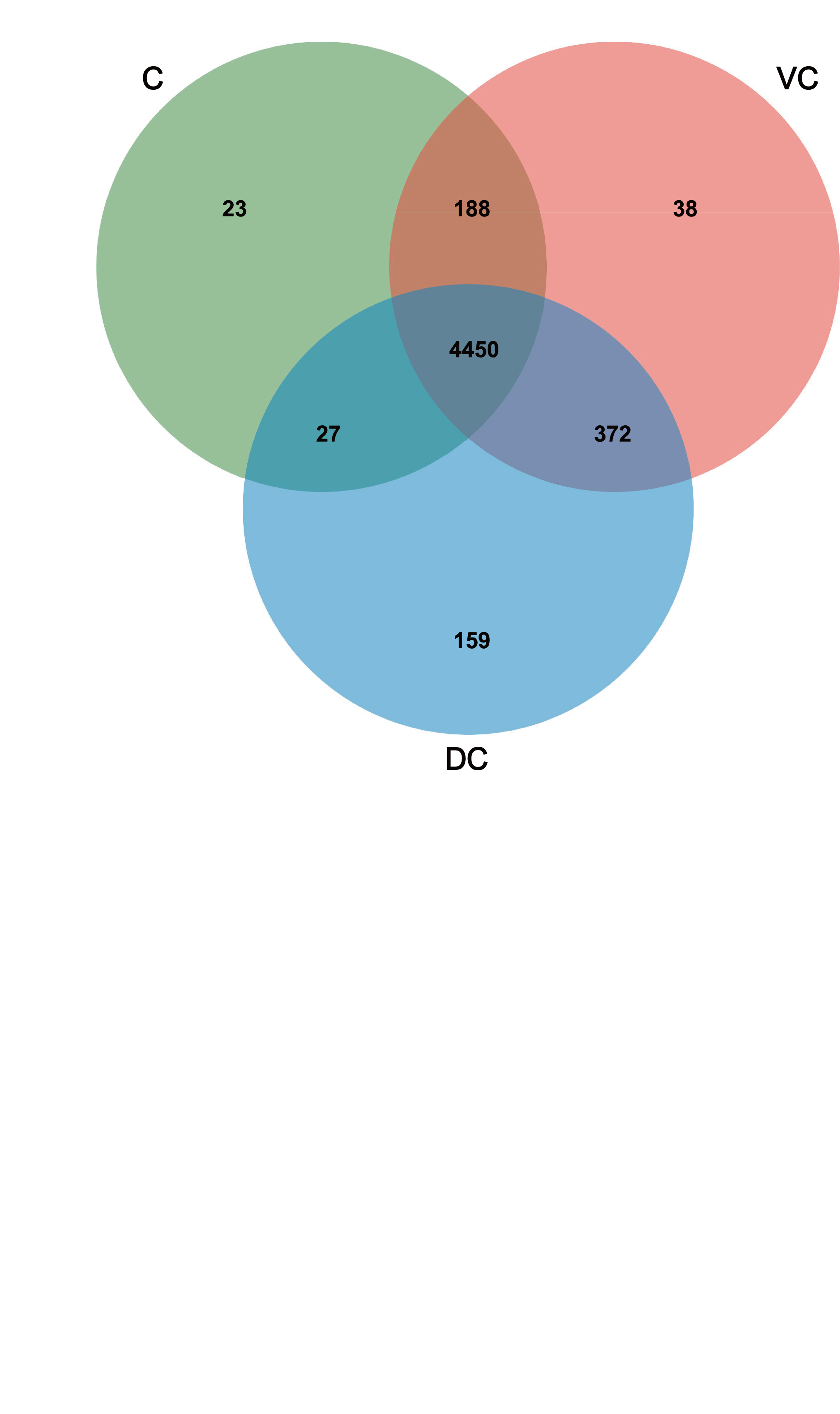


**Supplementary Figure 1.** Venn diagram depicting total, unique, and shared numbers of genera from the three segments of the donkey hindgut. C, cecum; VC, ventral colon; DC, dorsal colon.


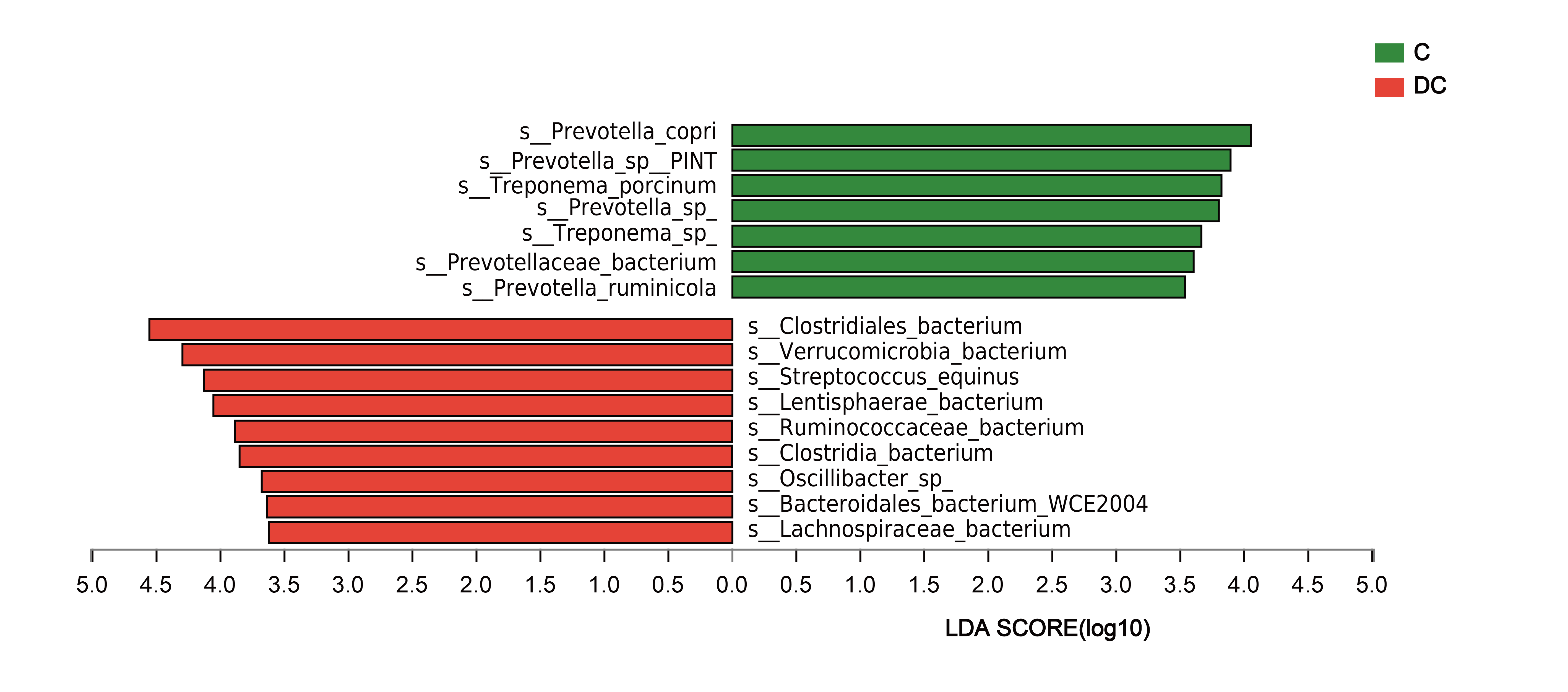


**Supplementary Figure 2**. Linear discriminant analysis (LDA) effect size (LEfSe) depicting the species that significantly differed between the C and DC groups (LDA score > 3.5, n=4). C, cecum; DC, dorsal colon.

**
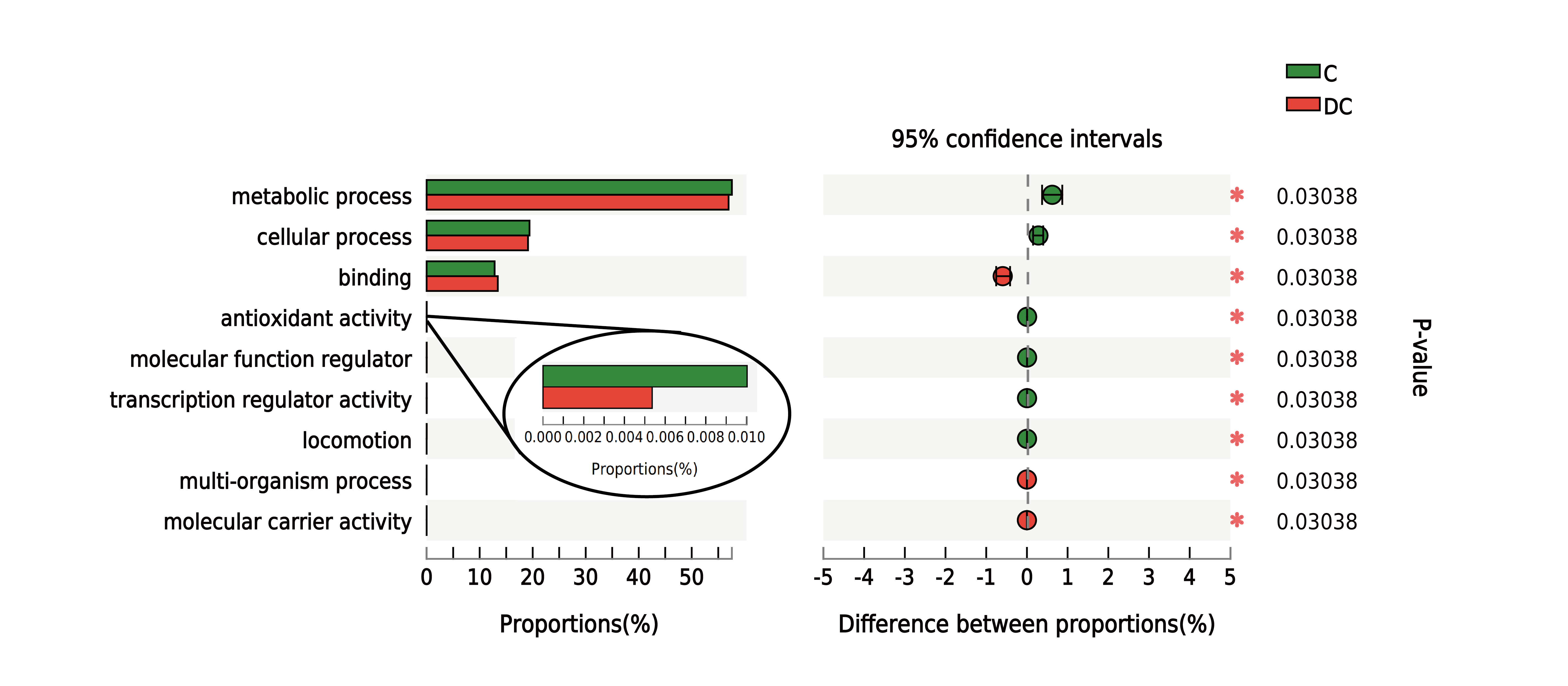
**

**Supplementary Figure 3.** Differential microbiota function based on the GO annotation between the C and DC groups at level 2. C, cecum; DC, dorsal colon.

**
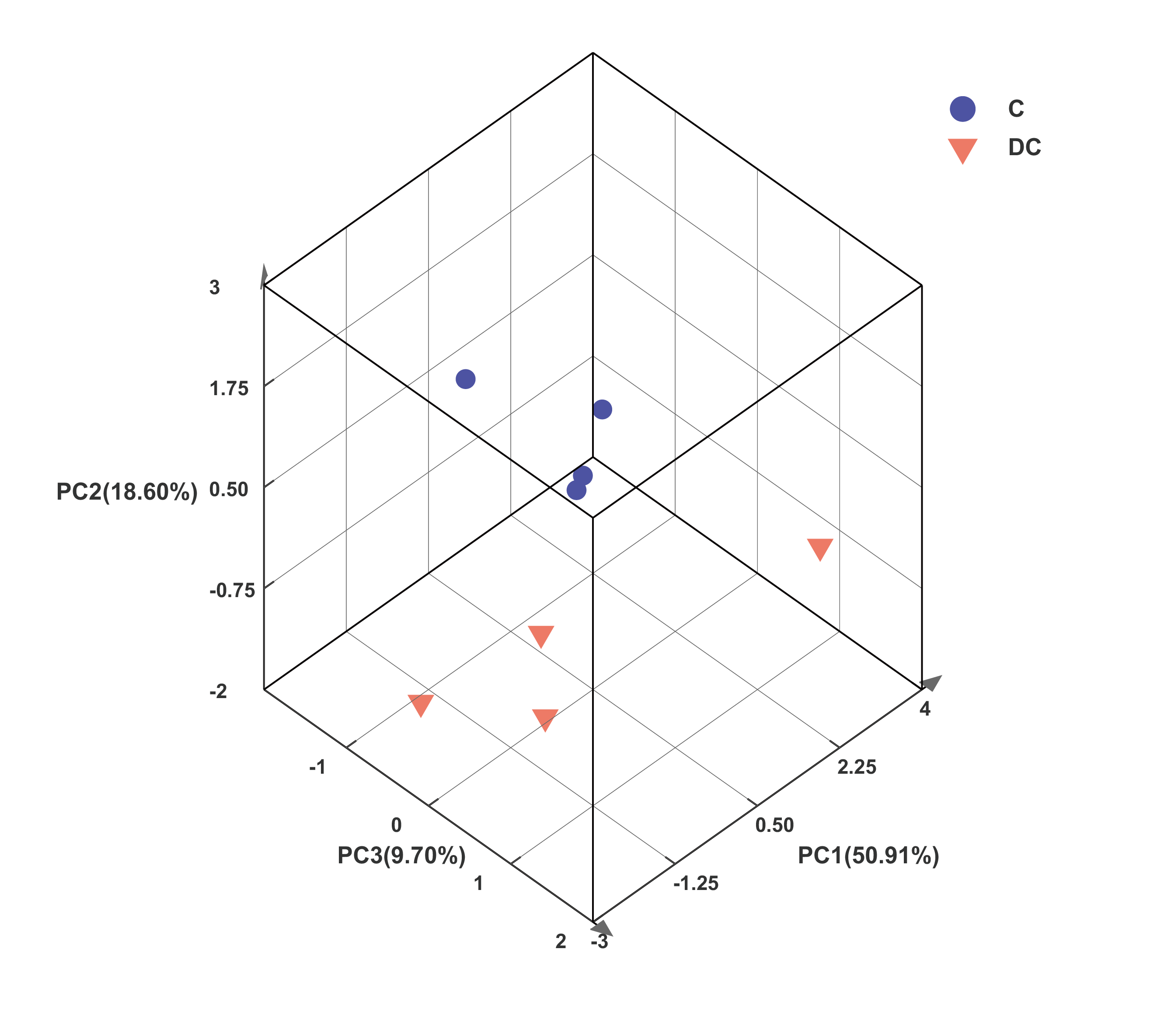
**

**Supplementary Figure 4.** Principal component analysis (PCA) based on transcriptome analysis of the C and DC groups. C, cecum; DC, dorsal colon.

**
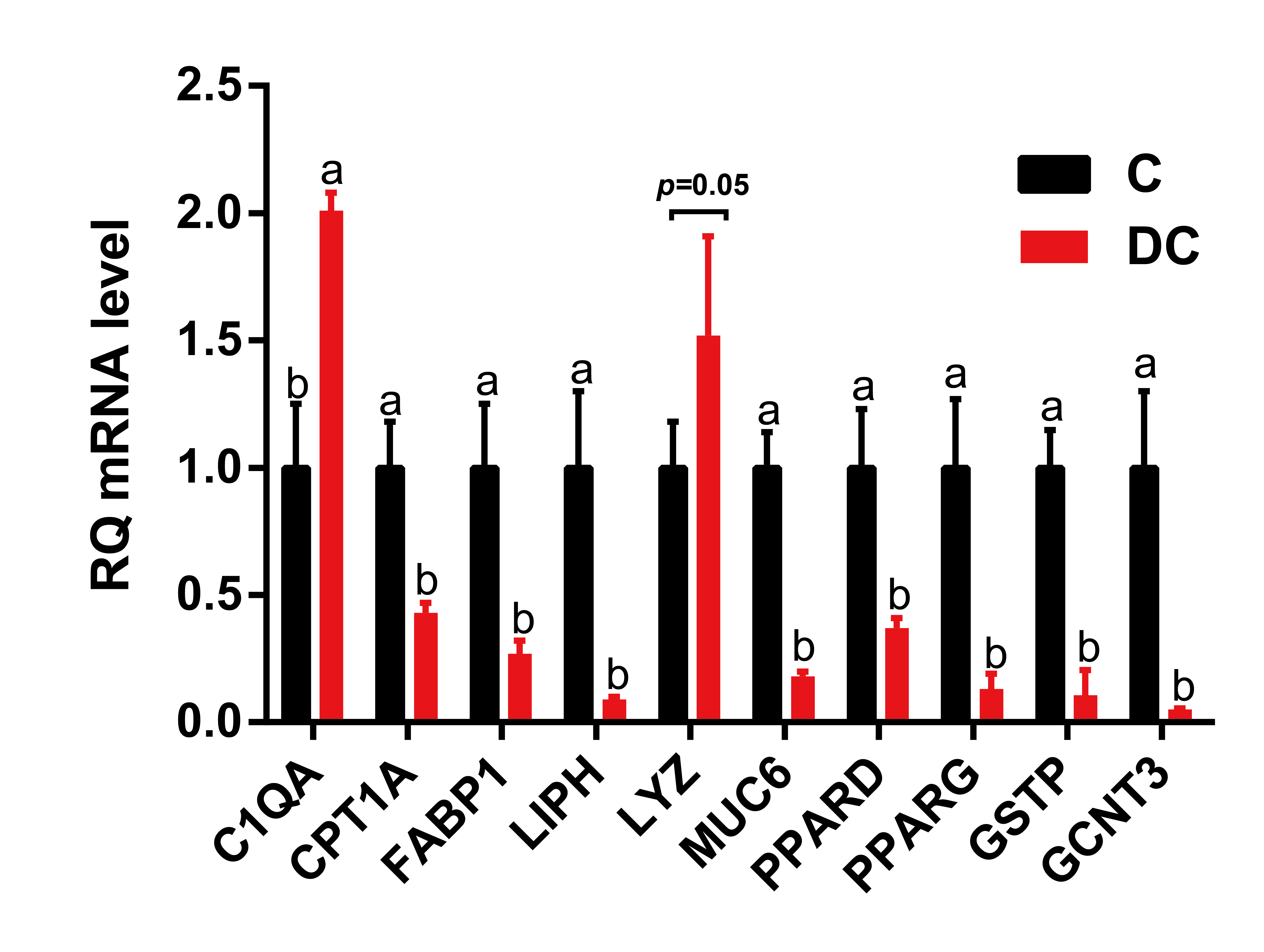
**

**Supplementary Figure 5.** qRT-PCR validation of RNA-seq data. Ten differentially expressed genes (DEGs) were randomly selected for qRT-PCR validation, while GAPDH was chosen as the reference gene. C, cecum; DC, dorsal colon. Data are expressed as the means ± SEM. ^a,b^ Means with different letters are significantly different, p < 0.05.

**
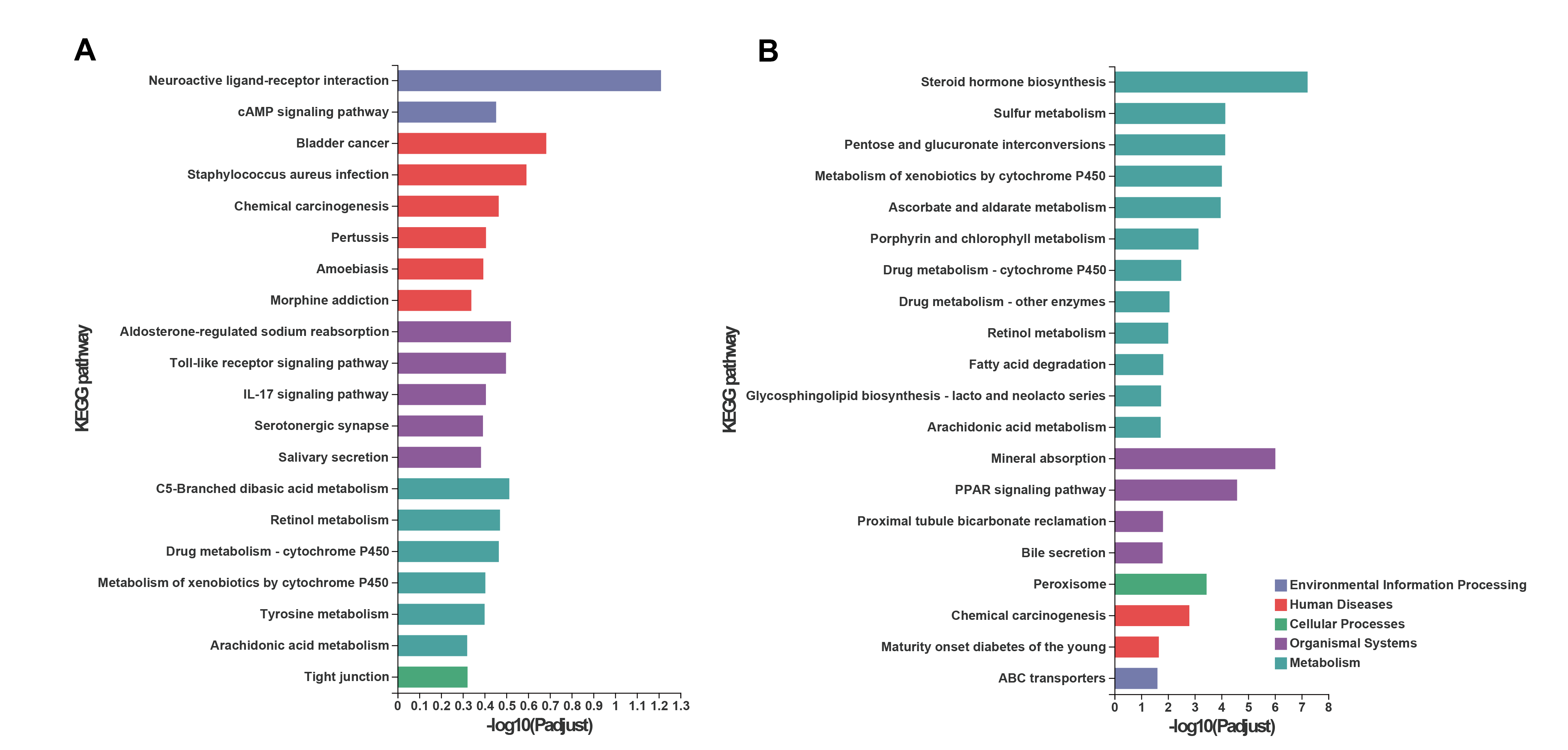
**

**Supplementary Figure 6.** Kyoto encyclopedia of genes and genomes (KEGG) pathway enrichment analysis of DEGs between the cecum and dorsal colon. (**A**) KEGG functional enrichment analysis of the upregulated DEGs. (**B**) KEGG functional enrichment analysis of the downregulated DEGs.

**
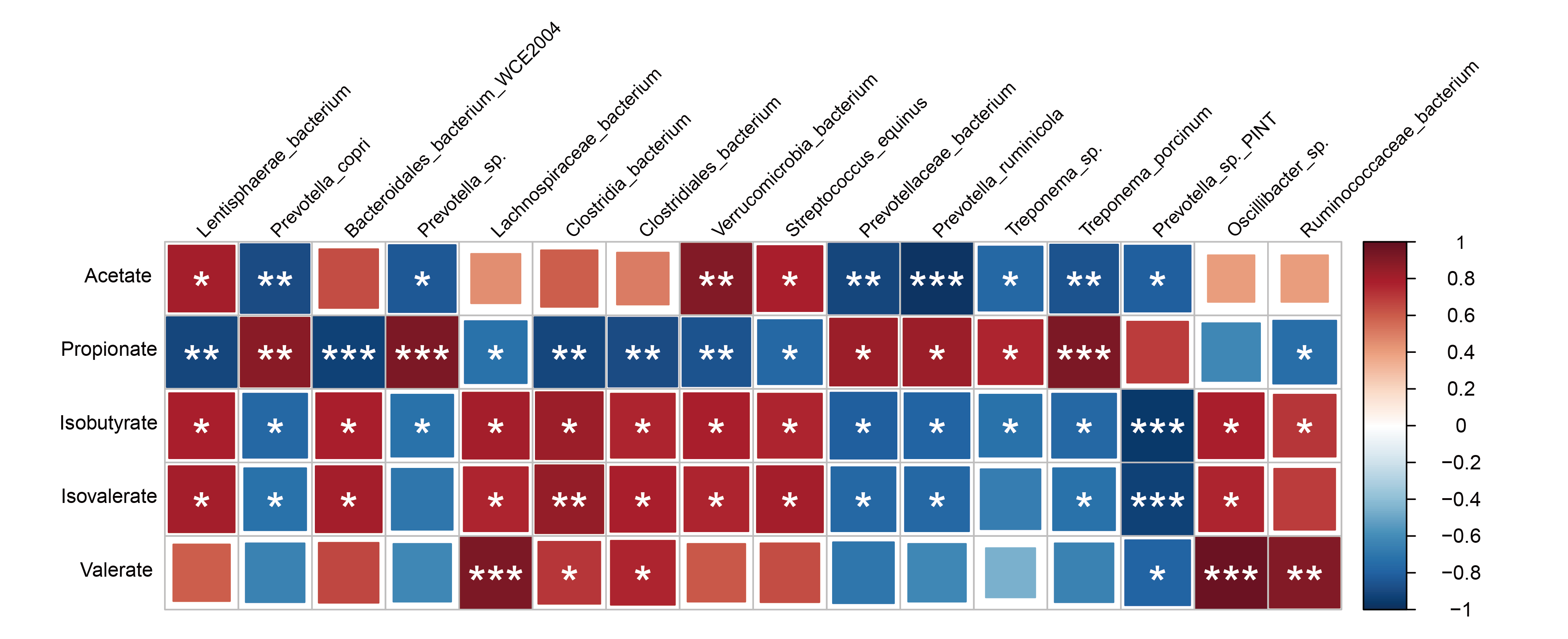
**

**Supplementary Figure 7.** Correlation between the SCFAs and the differentially enriched species. SCFAs, short-chain fatty acids.

**
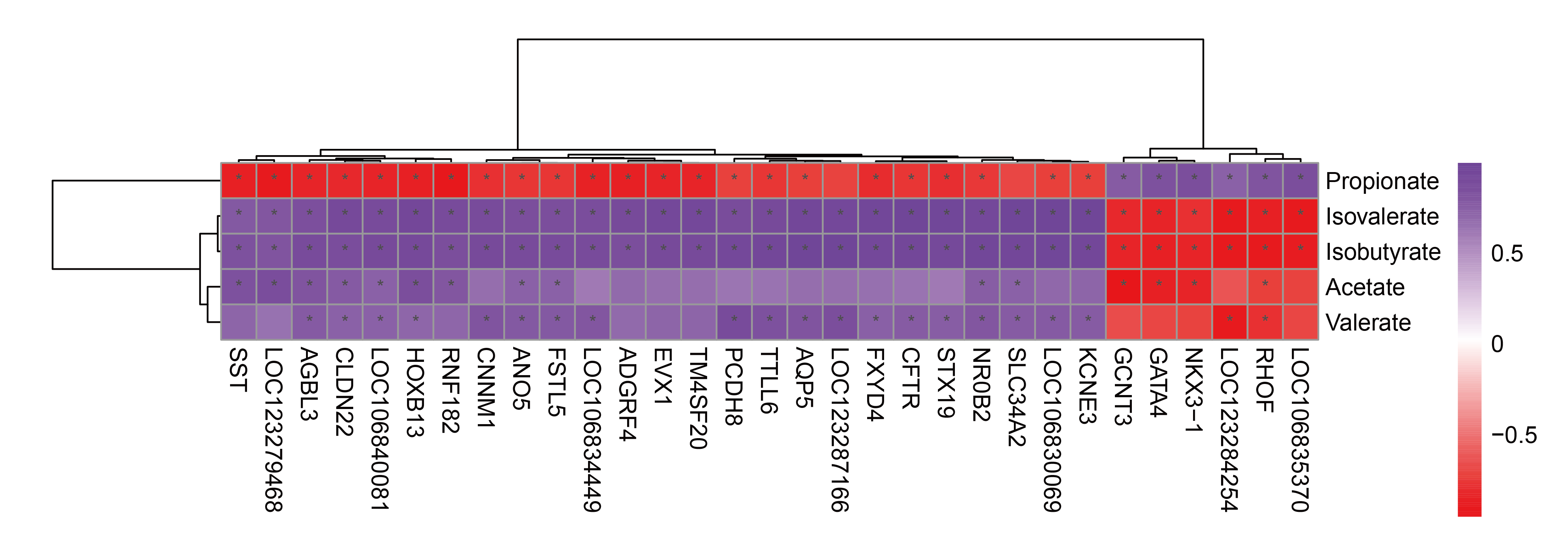
**

**Supplementary Figure 8.** Correlation between the SCFAs and DEGs. SCFAs, short-chain fatty acids.

## Supplementary Tables

**Supplementary Table 1** Basic information on sampled donkeys

| Ear Tag of Donkey | Breed | Age | Sex | Body Weight (Kg) | Body Length (cm) | Height of Withers (cm) | Rump Height (cm) | Rump Width (cm) | Rump Length (cm) | Circumference of Cannon Bone Girth (cm) | Thoracic Girth (cm) | Thoracic Width (cm) | Thoracic Depth (cm) |
| --- | --- | --- | --- | --- | --- | --- | --- | --- | --- | --- | --- | --- | --- |
| 1 | Dezhou Donkey | 2.5 | Male | 223 | 133.5 | 132 | 135 | 34 | 44 | 19 | 153 | 29 | 55.5 |
| 2 | Dezhou Donkey | 2.5 | Male | 239 | 130 | 130 | 132 | 36 | 41 | 17 | 139 | 31 | 52 |
| 3 | Dezhou Donkey | 2.5 | Male | 245 | 135 | 134 | 137 | 36 | 42 | 18 | 151 | 36 | 54 |
| 4 | Dezhou Donkey | 2.5 | Male | 210 | 126 | 130 | 131 | 35 | 40 | 17 | 143 | 29 | 54 |
| 5 | Dezhou Donkey | 2.5 | Male | 235 | 123 | 130 | 134 | 38 | 43 | 17.5 | 146 | 29 | 53 |
| 6 | Dezhou Donkey | 2.5 | Male | 239 | 137 | 129 | 131.5 | 40 | 43 | 17 | 137 | 31 | 55 |

**Supplementary Table 2** Primers for amplifying target genes with qPCR

| Target genes | Primers | Primer sequence (5’-3’) | Product size (bp) |
| --- | --- | --- | --- |
| FABP1 | FABP1 F | 5'TGAAGGCAGTCGGTATGT 3' | 139 |
|  | FABP1 R | 5' AAAGTGAACTCGTGGTGG 3' |  |
| LIPH | LIPH F | 5' TATCCCTGTGACTCCTACT 3' | 238 |
|  | LIPH R | 5' TGGACCCTCTTCTTATGT 3' |  |
| PPARG | PPARG F | 5' GGGGATGTCTCATAACGC 3' | 172 |
|  | PPARG R | 5' TTTGGTCAGTGGGAAGGA 3' |  |
| PPARD | PPARD F | 5' AGAGCACTCGCTTCCTTCC 3' | 265 |
|  | PPARD R | 5' AGCCTGATGCCTTGTCCC 3' |  |
| CPT1A | CPT1 F | 5' GCATCCGAGAAACATCAGC 3' | 157 |
|  | CPT1 R | 5' ACGACACGCCGTAACCAT 3' |  |
| LYZ | LYZ F | 5' ACTGTCCAGGGCAAGGTC 3' | 194 |
|  | LYZ R | 5' TTCCAGGATTGTAGTTTGTAGC 3' |  |
| C1QA | C1QA F | 5' GGTGTCCAAGTGGGATGTCTG 3' | 231 |
|  | C1QA R | 5' CCTCGGAGCCCTGGTAAAT 3' |  |
| MUC6 | MUC6 F | 5' CCTGAAGGCACCGTGTATG 3' | 151 |
|  | MUC6 R | 5' GGTGTACCTCTTCCCGTCAAA 3' |  |
| GSTP | GSTP F | 5' GGACGGAGACCTCACCCTGTA 3' | 177 |
|  | GSTP R | 5' TTGCCTGCCTCATAGTTGG 3' |  |
| GCNT3 | GCNT3 F | 5' CCATGAGGGAAACATCAGT 3 | 104 |
|  | GCNT3 R | 5' GAAGAATCCAAGGCAGGTC 3 |  |
| GAPDH | GAPDHF | 5' TGTCATCAACGGAAAGCC 3' | 183 |
|  | GAPDHR | 5' GCATCAGCAGAAGGAGCA 3' |  |

**Supplementary Table 3** Summary of metagenomics sequencing data for each sample

| Samples | Raw reads | Clean reads | Percent in raw reads (%) | Optimized reads | Percent in raw reads (%) |
| --- | --- | --- | --- | --- | --- |
| C1 | 94,552,964 | 93,265,824 | 98.64 | 59,843,250 | 63.29 |
| C2 | 91,324,444 | 89,962,192 | 98.51 | 69,244,282 | 75.82 |
| C3 | 89,324,984 | 88,322,024 | 98.88 | 70,343,140 | 78.75 |
| C4 | 89,329,740 | 88,306,148 | 98.85 | 56,545,292 | 63.30 |
| VC1 | 97,824,268 | 96,483,908 | 98.63 | 68,088,602 | 69.60 |
| VC2 | 86,409,162 | 85,037,558 | 98.41 | 58,371,592 | 67.55 |
| VC3 | 89,190,170 | 87,854,592 | 98.50 | 63,733,682 | 71.46 |
| VC4 | 84,392,148 | 83,003,936 | 98.36 | 60,490,744 | 71.68 |
| DC1 | 86,179,096 | 84,768,072 | 98.36 | 65,307,908 | 75.78 |
| DC2 | 85,739,296 | 84,300,008 | 98.32 | 66,255,682 | 77.28 |
| DC3 | 98,870,196 | 97,185,286 | 98.30 | 73,013,140 | 73.85 |
| DC4 | 99,262,128 | 97,735,326 | 98.46 | 83,166,034 | 83.78 |
| Total | 1,092,398,596 | 1,076,224,874 | —— | 794,403,348 | —— |
| Mean | 91,033,216 | 89,685,406 | 98.52 | 66,200,279 | 72.68 |
| SEM | 1,542,370 | 1,524,311 | 0.06 | 2,129,604 | 1.78 |

**Supplementary Table 4** Assembly statistics results of metagenomic datasets

| Samples | Contig number | Contigs bases (bp) | N50(bp) | N90(bp) | Max(bp) |
| --- | --- | --- | --- | --- | --- |
| C1 | 413,229 | 387,677,533 | 1,234 | 393 | 134,904 |
| C2 | 614,144 | 553,191,960 | 1,111 | 390 | 314,922 |
| C3 | 517,372 | 522,693,010 | 1,469 | 404 | 243,625 |
| C4 | 727,119 | 571,287,734 | 880 | 373 | 112,519 |
| VC1 | 583,436 | 541,694,749 | 1,202 | 395 | 287,175 |
| VC2 | 716,939 | 628,927,663 | 1,061 | 387 | 415,354 |
| VC3 | 717,073 | 649,722,575 | 1,143 | 390 | 442,601 |
| VC4 | 766,911 | 582,701,128 | 816 | 370 | 217,846 |
| DC1 | 725,323 | 628,505,643 | 1,049 | 383 | 177,416 |
| DC2 | 861,054 | 686,002,556 | 892 | 374 | 415,354 |
| DC3 | 837,504 | 719,251,892 | 1,018 | 385 | 262,799 |
| DC4 | 997,800 | 778,838,413 | 853 | 372 | 302,277 |

**Supplementary Table 5** Comparison of SCFA levels (μg/g) between the C and DC groups

| **Index** | **C** | **DC** | **P value** |
| --- | --- | --- | --- |
| Acetate | 513.18±5.04^b^ | 601.44±36.34^a^ | 0.037 |
| Propionate | 667.75±58.82^a^ | 308.88±22.84^b^ | 0 |
| Isobutyrate | 9.71±1.17^b^ | 82.53±5.09^a^ | 0 |
| Butyrate | 247.02±23.57 | 229.65±19.18 | 0.58 |
| Isovalerate | 9.04±1.26^b^ | 86.93±4.92^a^ | 0 |
| Valerate | 34.40±3.78^b^ | 74.83±4.42^a^ | 0 |
| SCFAs | 1484.89±83.51 | 1398.02±73.90 | 0.454 |

Note: n=6, Mean±SEM. SCFAs: Short chain fatty acids; C: Cecum; DC: Dorsal colon. ^a , b^ Means within the same line with different superscript are significantly different, p< 0.05.
